# Microtubules and Dynein Regulate Human Neutrophil Nuclear Volume and Hypersegmentation During *H. pylori* Infection

**DOI:** 10.3389/fimmu.2021.653100

**Published:** 2021-03-22

**Authors:** Stephanie L. Silva-Del Toro, Lee-Ann H. Allen

**Affiliations:** ^1^Inflammation Program of the University of Iowa, Iowa City, IA, United States; ^2^Immunology Graduate Program of the University of Iowa, Iowa City, IA, United States; ^3^Department of Internal Medicine, University of Iowa, Iowa City, IA, United States; ^4^Department of Microbiology and Immunology, University of Iowa, Iowa City, IA, United States; ^5^Iowa City VA Healthcare System, Iowa City, IA, United States

**Keywords:** neutrophils, plasticity, hypersegmentation, microtubules, dynein, lamin B receptor

## Abstract

Neutrophils (also called polymorphonuclear leukocytes, PMNs) are heterogeneous and can exhibit considerable phenotypic and functional plasticity. In keeping with this, we discovered previously that *Helicobacter pylori* infection induces N1-like subtype differentiation of human PMNs that is notable for profound nuclear hypersegmentation. Herein, we utilized biochemical approaches and confocal and super-resolution microscopy to gain insight into the underlying molecular mechanisms. Sensitivity to inhibition by nocodazole and taxol indicated that microtubule dynamics were required to induce and sustain hypersegmentation, and super-resolution Stimulated Emission Depletion (STED) imaging demonstrated that microtubules were significantly more abundant and longer in hypersegmented cells. Dynein activity was also required, and enrichment of this motor protein at the nuclear periphery was enhanced following *H. pylori* infection. In contrast, centrosome splitting did not occur, and lamin B receptor abundance and ER morphology were unchanged. Finally, analysis of STED image stacks using Imaris software revealed that nuclear volume increased markedly prior to the onset of hypersegmentation and that nuclear size was differentially modulated by nocodazole and taxol in the presence and absence of infection. Taken together, our data define a new mechanism of hypersegmentation that is mediated by microtubules and dynein and as such advance understanding of processes that regulate nuclear morphology.

## Introduction

Neutrophils or polymorphonuclear leukocytes (PMNs) are the most abundant white blood cell in circulation, are the first leukocytes to be recruited to sites of infection, and are essential for innate host defense (1–3). PMNs are also short-lived and terminally differentiated and are notable for their peculiar, segmented nuclei comprised of three or four interconnected lobes (2, 4). Until recently, PMNs were thought to be a homogeneous population of cells with a singular phenotype. However, this notion has been fundamentally changed by the discovery of PMN plasticity and the characterization of different cell populations that differ in maturation state, density, functional properties and nuclear morphology (5–8). From tumor-associated neutrophils to professional antigen presenting cells involved in adaptive immunity, to bacterial and parasitic infections and sepsis as well as autoimmune disease, a spectrum of pro-inflammatory, regulatory and suppressive PMNs have been described. Hypersegmented PMN subsets were first noted in the murine tumor microenvironment and more recently have been identified in patients with lung and ovarian cancer, obstructive lung disease, juvenile inflammatory arthritis, hyperthermia, and during experimental endotoxemia (9–15). Previously thought to occur only in response to *in vivo* cues, we discovered that infection of human neutrophils with the ulcer and gastric cancer-causing Gram-negative bacterium *Helicobacter pylori* can induce N1-like subtype differentiation of human neutrophils *in vitro* that is notable for profound nuclear hypersegmentation, proinflammatory cytokine secretion, a CD62L^dim^, CD16^bright^, CD11b^bright^, CD66b^bright^, CD63^bright^ surface phenotype and a 72 h lifespan (16). More recently, Ohms et al. (17) also attempted *in vitro* polarization of human neutrophils, but their analysis did not include examination of nuclear morphology.

Hypersegmentation is defined as 5% of PMNs with five nuclear lobes or any single cell with six lobes or more and was described first in the context of folate or vitamin B12 deficiency which impairs DNA synthesis during neutrophil development in the bone marrow (18–20). Conversely, the mechanisms that mediate hypersegmentation of PMN subsets are unknown. During *H. pylori* infection, hypersegmentation is not linked to folate or vitamin B12, but does require direct infection and both host and bacterial transcription and protein synthesis (16). Herein, we used this tractable system to gain fundamental new insight into the mechanisms that control neutrophil nuclear morphology.

## Materials and Methods

### Cultivation of *H. pylori*

*H. pylori* strain NCTC11637 (ATCC 43504) was grown for 24 h under humidified, microaerophilic conditions (5% O_2_, 10% CO_2_, 85% N_2_) at 37°C on trypticase soy agar (Difco, Franklin Lakes, NJ) containing 5% defibrinated sheep blood (Remel, Lenexa, KS) and supplemented with 5 mg/L cefsulodin, 5 mg/L trimethoprim, and 10 mg/L vancomycin (21). Bacteria were harvested from plates, washed twice in PBS containing 1 mM glucose and quantified by measuring absorbance at 600 nm.

### Ethics Statement

Heparinized venous blood was obtained from healthy adult donors who provided written informed consent using procedures approved by the Institutional Review Board of the University of Iowa (IRB #200307026).

### PMN Isolation and Bacterial Infection

PMNs were isolated by dextran sedimentation and Ficoll-Hypaque gradient separation followed by hypotonic lysis of erythrocytes (22). Neutrophils were routinely 95-98% pure with eosinophils as the major contaminant. Cells were resuspended at 2 × 10^6^ cells/mL in Hepes-buffered RPMI-1640 medium (Lonza, Walkersville, MD) supplemented with 10% heat-inactivated FBS (HyClone Laboratories, Pittsburgh, PA) and 2 mM L-glutamine (Lonza, Walkersville, MD) and then incubated in suspension at 37°C under microaerophilic conditions in the presence or absence of *H. pylori* at a multiplicity of 5 bacteria/cell. Replicate experiments utilized PMNs from different donors.

### Inhibitor Treatment

Drugs at the following final concentrations were added at time zero unless otherwise indicated: nocodazole (Calbiochem, San Diego, CA) at 10 μM, taxol (Sigma, Burlington, MA) at 1 μM, and ciliobrevin D (EMD Millipore Sigma, Burlington, MA) at 20 μM.

### Hema-3 Staining and Nuclear Morphology Analysis

PMNs were attached to coverslips by cytocentrifugation and immediately stained using Hema-3 Stat Pack reagents (Thermo Fisher Scientific, Waltham, MA) (16, 23). Nuclear morphology was analyzed by light microscopy (Zeiss Axioplan 2 with Axiovision software, Carl Zeiss Inc., Thornwood, NY) as we described (16).

### Immunofluorescence and Confocal Microscopy

Cells were attached to acid washed coverslips coated with human serum as we described (24, 25) and then gently washed with PBS prior to fixation, with conditions optimized for detection of different antigens. Our standard conditions were used to detect lamin B receptor (LBR) and lamin B1 (25). In brief, cells were fixed in 10% formalin for 15 min, permeabilized with cold methanol-acetone (1:1) for 5 min, rinsed with PBS containing 0.5 mg/mL NaN_3_ and 5 mg/mL BSA (PAB) and then blocked overnight in PAB containing 10% horse serum. To detect dynein and microtubules (MTs), cells were fixed and permeabilized as described by Ding et al. (26), with minor modifications. Specifically, cells were fixed in 4% paraformaldehyde (PFA) immediately followed by addition of 0.2% glutaraldehyde, for a final concentration of 2 and 0.1%, respectively, in PBS containing 0.002% NaN_3_ (PBSa) and incubated for 15 min at room temperature. After a PBSa wash, cells were permeabilized in 0.5% SDS in PBSa, and then blocked as described above. To visualize the ER, cells were fixed with 4% PFA for 15 min and then permeabilized with 0.02% Triton X-100 for 5 min, both at room temperature.

Blocked cells were stained with a 1/1,000 dilution of affinity-purified goat anti-*H. pylori* polyclonal antibodies (pAb) (SeraCare, Milford, MA), 1/200 mouse anti-γ-tubulin mAb (clone GTU-88, Abcam, Cambridge, MA), 1/500 rabbit anti-α-tubulin (EP1332Y, Abcam, Cambridge, MA), 1/100 mouse anti-dynein (74.1, Abcam, Cambridge, MA), 1/100 mouse anti-protein disulfide-isomerase (PDI) (RL77, Thermo Fisher Scientific, Waltham, MA), 1/1,000 rabbit anti-Lamin B Receptor (E398L, Abcam, Cambridge, MA), or 1/1,000 pAb rabbit anti-lamin B1 (Abcam, Cambridge, MA) as we described (25). Affinity-purified F(ab')_2_ secondary antibodies were obtained from Jackson ImmunoResearch Laboratories (West Grove, PA) and used at 1/200 dilution. In each case, specificity of staining was determined by omission of primary antibodies. Background signals obtained from secondary antibodies alone are shown in Supplementary Figure 1.

Coverslips were mounted to slides using Prolong Diamond Antifade mountant with DAPI (Invitrogen, Carlsbad, CA) and analyzed using a Zeiss LSM880 confocal microscope, with a 1 AU pinhole aperture and utilizing the Zeiss Zen acquisition software. Z-stacks were obtained of each cell and 3D reconstructions and processing used Oxford Bitplane Imaris Software (version 9.2.1). At least 100 cells per condition were analyzed for each condition and donor.

### Stimulated Emission Depletion Microscopy (STED)

PMNs were attached to serum-coated, acid washed #1.5 German glass coverslips (Electron Microscopy Sciences, Hatfield, PA), fixed and permeabilized as described above and stained with 1/100 goat anti-rabbit LBR (clone E398L, Abcam, Cambridge, MA) or 1/25 purified rat anti-tyrosinated α-tubulin (clone YL1/2, Millipore Sigma, Burlington, MA) as above, except incubation with primary antibodies was increased to 2 h. Z-stack images were acquired using a Leica SP8 STED Super Resolution Microscope and LAS X software (Leica Microsystems, Buffalo Grove, IL). A resolution of 40-50 nm was obtained after processing using Huygens Professional Software deconvolution wizard (version 19.04) set to a maximum of 40 iterations. Volume, area and microtubule quantifications were done using Oxford Bitplane Imaris Software (version 9.2.1) surface and filament extensions.

### Flow Cytometry

After incubation in Intracellular Fixation Buffer (Thermo Fischer Scientific, Waltham, MA) for 30 min at room temperature and permeabilization with cold 100% methanol on ice for 30 min, cells were stained with FITC-conjugated anti-LBR mAb (clone e398L, Abcam, Cambridge, MA) diluted 1/50 in blocking buffer (1% FBS in PBS). After washing, cells were resuspended in PBS and 10,000 events/sample were acquired using an Accuri C6 Cytometer (BD Biosciences, East Rutherford, NJ) followed by FlowJo V10 analysis.

### Western Blotting

Whole cell lysates were prepared as we described (27). Proteins were separated on NuPAGE™ 4-12% Bis-Tris protein gels (Invitrogen, Carlsbad, CA) and then transferred to polyvinylidene fluoride membranes using a Trans-Blot Turbo (Bio-Rad, Hercules, CA). Blocked membranes were probed with 1/1,000 anti-α-tubulin (EP1332Y, Abcam, Cambridge, MA) or 1/1,000 anti-dynein (74.1, Abcam, Cambridge, MA) and then stripped and re-probed with 1/2,000 anti-GAPDH (6C5, Calbiochem, San Diego, CA). HRP-conjugated secondary antibodies were from GE Healthcare (Chicago, IL) and Bio-Rad (Hercules, CA) and were used at 1/2,000. Bands were detected using West Femto reagents (Thermo Fischer Scientific, Waltham, MA) with Li-Cor Odyssey imaging and Image Studio Software analysis.

### Statistical Analysis

All experiments were performed at least three times. Analyses utilized GraphPad Prism 7 or 8 with *P* < 0.05 considered statistically significant. See figure legends for details.

## Results

### Microtubule Dynamics Are Required to Induce Hypersegmentation

**Figure 1:**
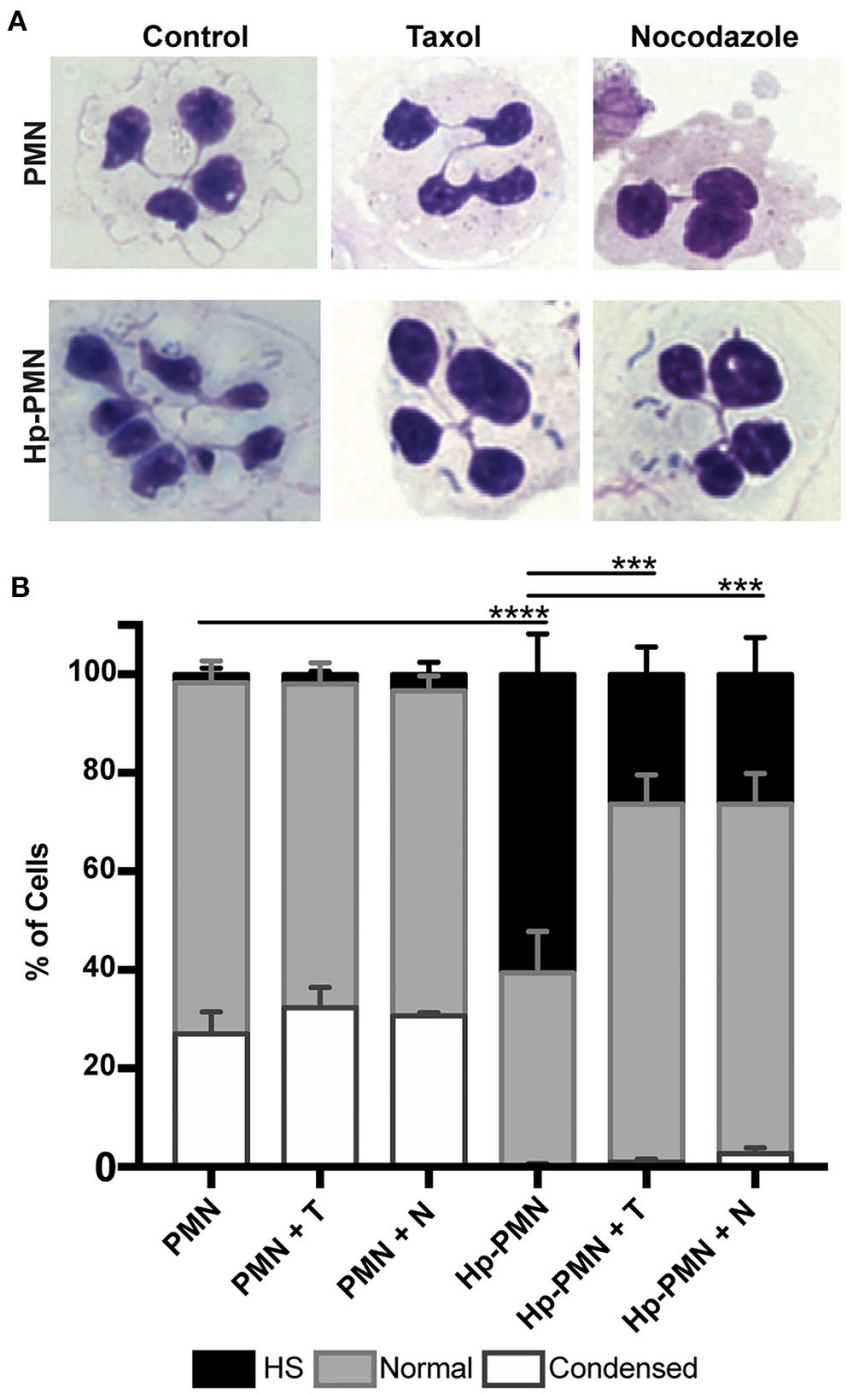
Microtubule dynamics are required for *H. pylori*-induced hypersegmentation. Human PMNs were infected with *H. pylori* (Hp) and/or treated with taxol (T) or nocodazole (N) for 24 h. Cells were attached to coverslips by cytocentrifugation and immediately stained with Hema-3 reagents. **(A)** Representative light microscopy images of treated and control PMNs (x1,000 original magnification). **(B)** Nuclei were scored as hypersegmented (HS, 5, or more lobes), normal (3-4 lobes), or condensed (1-2 lobes). Pooled data are the mean + standard error of the mean from three independent experiments. ***P ≤ 0.001 and ****P ≤ 0.0001 by two-way ANOVA with Tukey's multiple comparison test indicate differences in hypersegmentation.

To test the hypothesis that MTs are required for *H. pylori*-induced nuclear hypersegmentation PMNs were treated with taxol or nocodazole at time zero in the presence and absence of bacteria and nuclear morphology was assessed at 24 h using Hema-3 staining and light microscopy. As expected, the majority of uninfected PMNs had normal lobed nuclei and a subset of cells had condensed spherical nuclei indicative of progression to apoptosis (16, 23). In marked contrast, the majority of infected cells (Hp-PMNs) contained as many as 19 nuclear lobes and are defined as hypersegmented (16). However, when MTs were bundled by taxol or depolymerized by nocodazole (confirmed in Supplementary Figure 2A) hypersegmentation was significantly inhibited (Figures 1A,B). Based on these data, we conclude that MT dynamics were required for *H. pylori*-induced hypersegmentation but not for sustained lobulation of mature neutrophil nuclei or for the nuclear condensation that accompanies progression to apoptosis.

### Microtubules Are More Abundant Following *H. pylori* Infection and Microtubule Dynamics Are Required for Sustained Hypersegmentation

**Figure 2:**
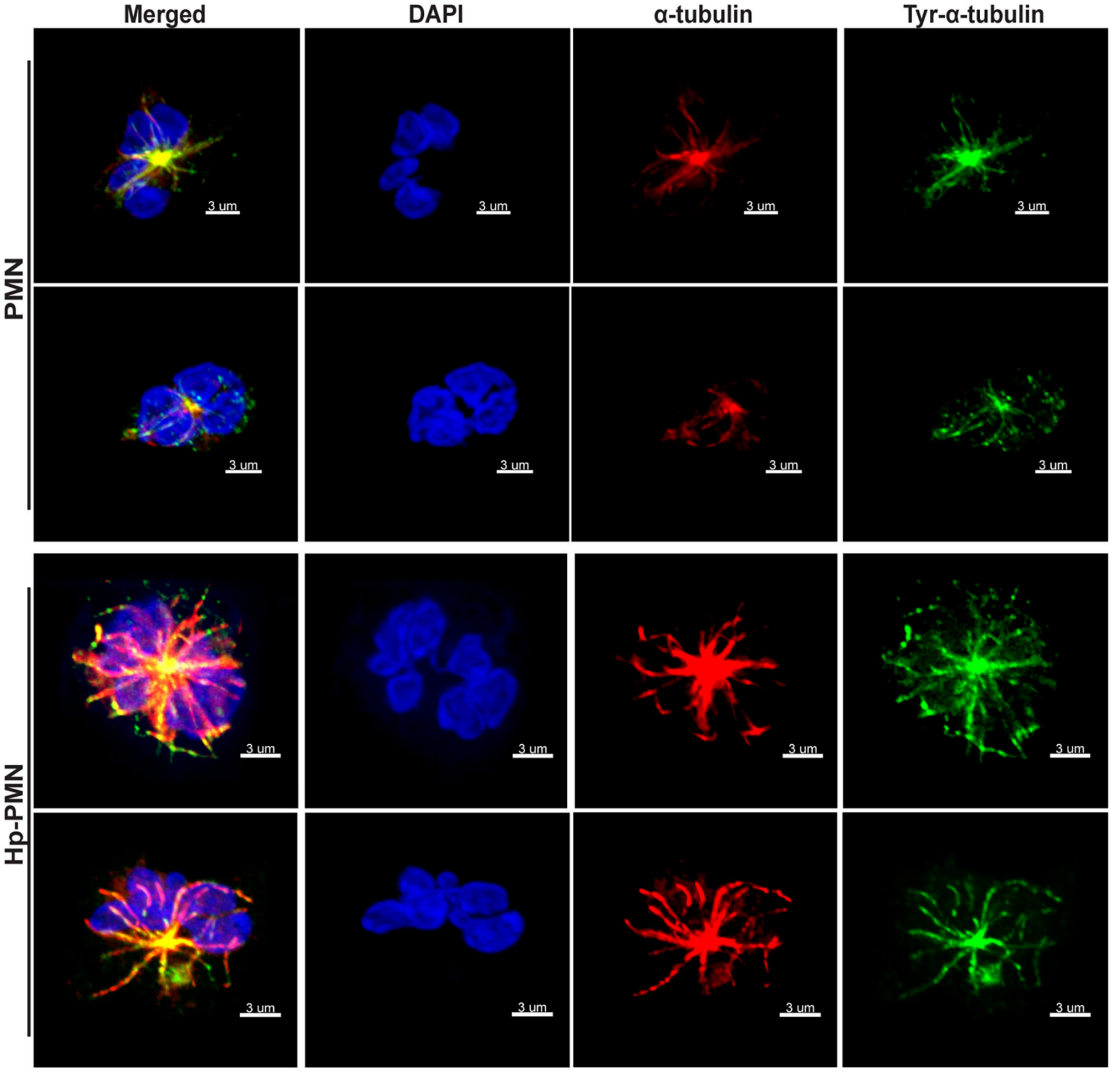
MTs appear to be more abundant in infected PMNs and tyrosination is prevalent throughout MTs independent of infection. Control and infected PMNs were analyzed by confocal microscopy at 24 h after triple-staining to detect DNA (DAPI, blue), α-tubulin (Alexa Fluor 549, red), and tyrosinated α-tubulin (FITC, green). Images are representative 3D reconstructions of confocal Z-stacks from three independent experiments. Scale bar = 3 μm (x1,000 original magnification).

Next, we used immunofluorescence and confocal microscopy to detect MTs in control and infected PMNs after 24 h at 37°C. Staining with anti-α-tubulin antibodies showed that MTs radiate from the perinuclear microtubule organizing center (MTOC) in both control and infected cells, and similar results were obtained using antibodies specific for tyrosinated α-tubulin, a marker of dynamic MTs (28) (Figure 2). Staining for the MTOC marker γ-tubulin (29) demonstrated that each cell contained a single focus for MT polymerization (Supplementary Figure 2B), excluding centrosome splitting as a mechanism contributing to hypersegmentation in our system (30).

**Figure 3:**
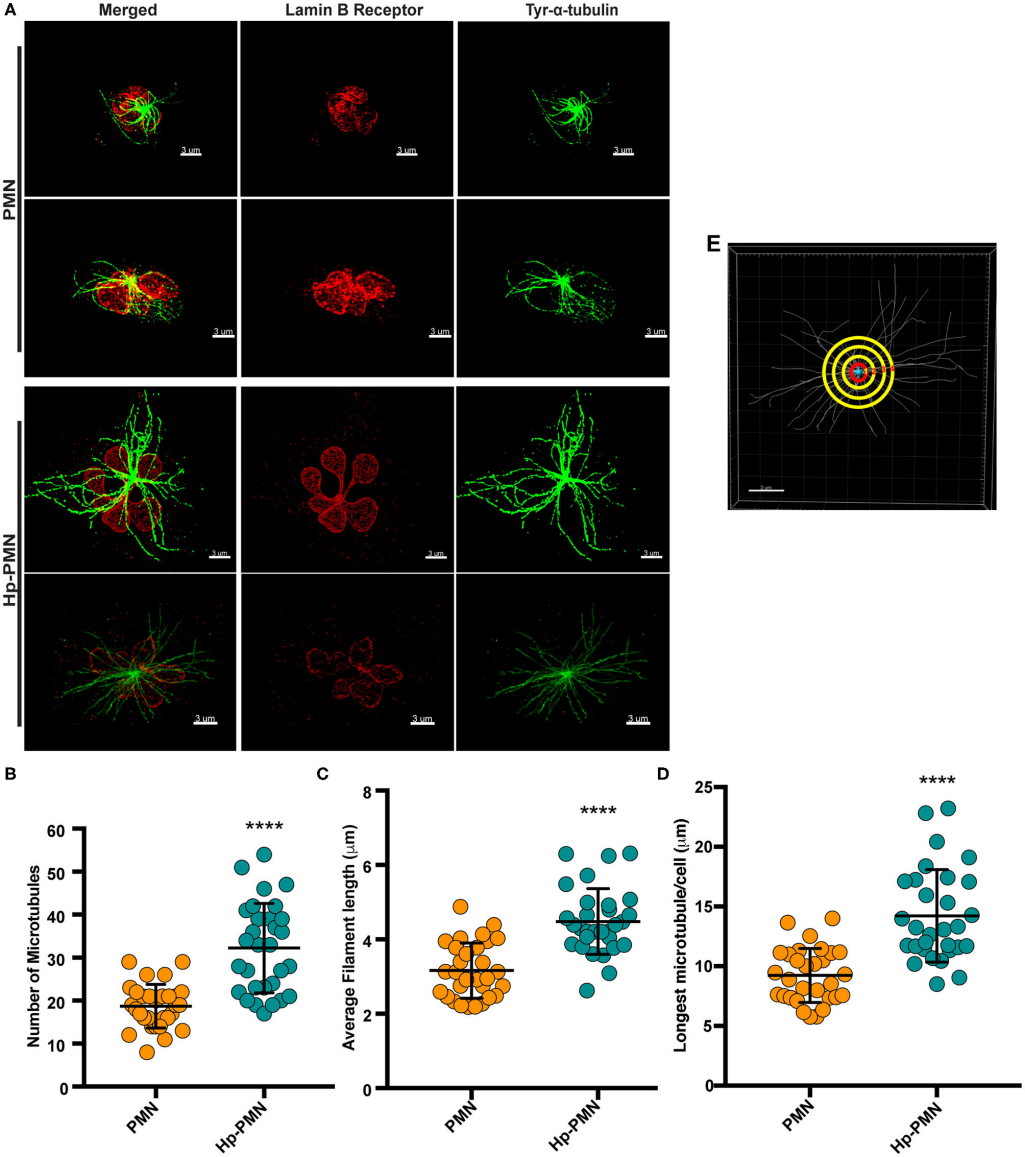
MTs are more abundant and longer in infected PMNs. Infected and control PMNs were processed for analysis by STED at 24 h. **(A)** Representative 3D reconstructions of STED Z-stack images. Lamin B receptor (LBR) was detected using TRITC-conjugated secondary antibodies (red) and tyrosinated α-tubulin was detected using FITC-conjugated secondary antibodies (green). Scale bar = 3 μm (x1,000 original magnification). **(B–E)** MT length and abundance were quantified for 10 cells per donor and condition in each experiment. Graphs show the number of MTs radiating from the MTOC **(B)**, average filament length **(C)**, and longest MT in each cell **(D)**. Data for individual cells (symbols) are shown along with the mean and standard deviation (*n* = 3). A Welsh *t*-test was done to determine significance. ****P ≤ 0.0001. **(E)** Representation of MT analysis. The red circle denotes 1 μm from the MTOC. Tubules that intersect this circle were counted **(B)**. MTs in each image were traced using the Autodepth Filament feature of Imaris **(C,D)**.

As our confocal data suggested that MTs may be more abundant in Hp-PMNs (Figure 2), we analyzed these structures in detail using super-resolution STED microscopy. Representative images are shown in Figure 3A, along with quantitation of MT number (Figure 3B) and length (Figures 3C,D) determined using Imaris software (Figure 3E, schematic). These data demonstrate that MTs were more abundant and longer in Hp-PMNs, results that are also apparent by comparison of Supplementary Videos 1, 2. Nevertheless, the overall abundance of α-tubulin in control and infected cells was unchanged as judged by western blotting (data not shown).

**Figure 4:**
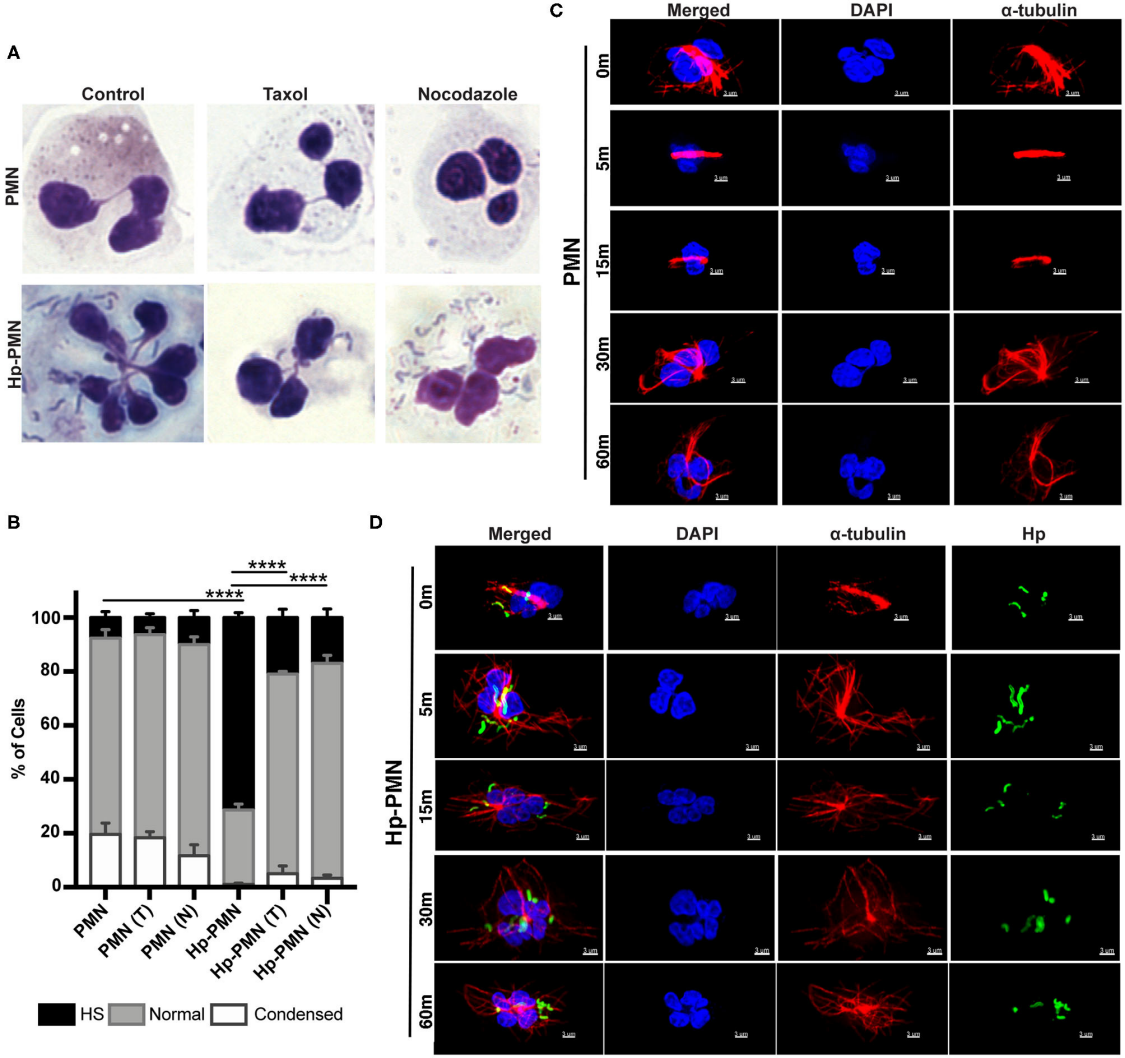
MT dynamics are required to sustain *H. pylori*-induced hypersegmentation. **(A,B)** Infected and uninfected PMNs were treated with taxol (T) or nocodazole (N) at 18 h. Representative images of Hema-3-stained cells are shown in **(A)** and pooled data are shown in **(B)**. Nuclei were scored as hypersegmented (HS), normal or condensed as in Figure 1. Graph shows the mean + standard error of the mean, *n* = 3. Significant differences in hypersegmentation were detected using two-way ANOVA and Tukey's multiple comparison test. ****P ≤ 0.0001. **(C,D**) PMNs were treated with taxol in the presence and absence *H. pylori* at time zero. Taxol was removed at 18 h and the kinetics of MT rearrangement over 5-60 min at 37°C was analyzed using confocal microscopy. Representative images of uninfected PMNs **(C)** and infected PMNs **(D)** that were stained to detect DNA (DAPI, blue) and α-tubulin (Alexa Fluor 549, red) alone or together with *H. pylori* (Alexa Fluor 488, green), *n* = 3. Scale bar = 3 μm (x1,000 original magnification).

To determine if hypersegmentation was reversible, we added taxol or nocodazole to PMNs at 18 h post-infection (hpi), and then processed cells for microscopy 6 h later (at 24 hpi). As shown in Figure 4, ~70% of Hp-PMN were hypersegmented by 18 hpi, but only a small fraction of cells retained this morphology after drug treatment, and as such were indistinguishable from the uninfected controls (Figures 4A,B).

To gain further insight into the kinetics of MT rearrangements, uninfected and infected cells that had previously been treated with taxol were analyzed by microscopy 5-60 min after drug washout. In uninfected PMNs, MTs remained bundled 15 min after taxol was removed and had only partially rearranged after 30–60 min (Figure 4C). Conversely, MTs rearranged very rapidly in Hp-PMN, as bundled MTs were diminished or absent by 5 min and normal MT asters radiating from the MTOC were restored by 15 min (Figure 4D). These data demonstrate that hypersegmentation was reversible, and that MT dynamics were required to sustain as well as induce this atypical nuclear morphology.

### Dynein Localization and Activity in Hypersegmentation

**Figure 5:**
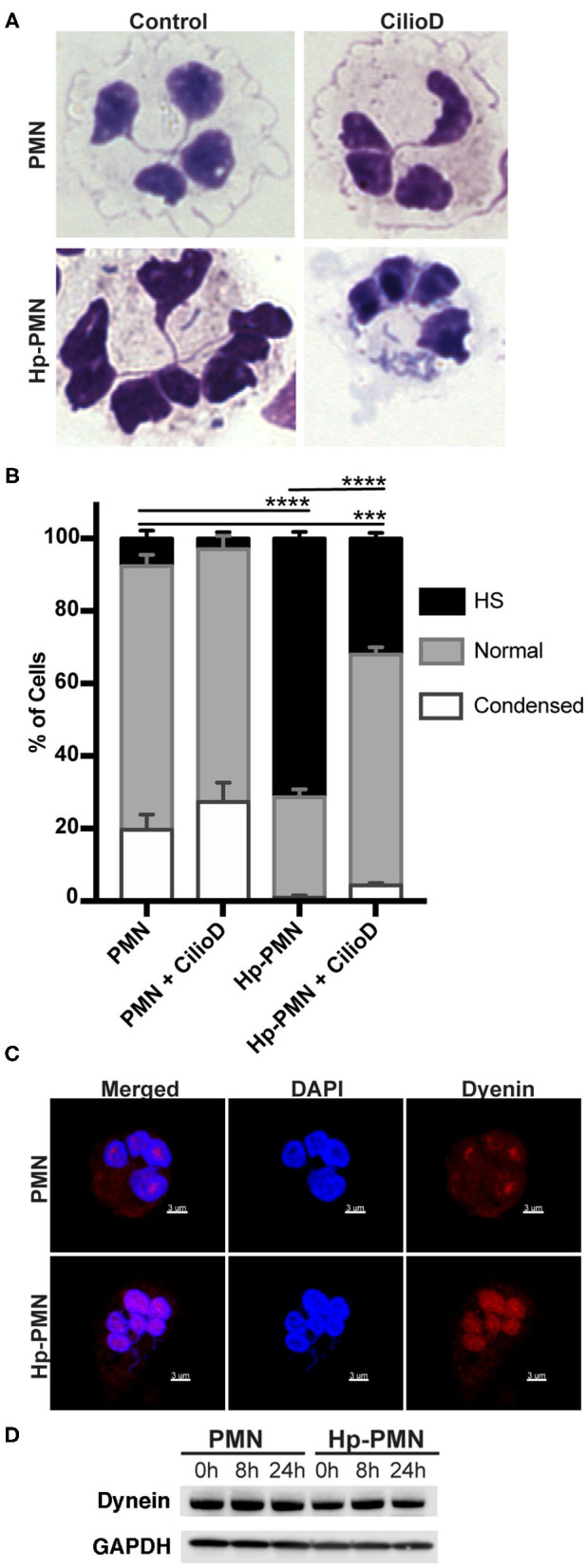
Dynein-mediated trafficking is required for hypersegmentation. **(A,B)** Cells were treated with 20 μM Ciliobrevin D for 24 h in the presence and absence of *H. pylori*. Nuclear morphology was scored by light microscopy after Hema-3 staining. Representative images **(A)** and pooled data **(B)** are shown. Graphs indicate the mean + standard error of the mean. Significant differences in hypersegmentation (HS) were determined using two-way ANOVA and Tukey's multiple comparison test. ***P ≤ 0.001, ****P ≤ 0.0001, *n* = 3. **(C)** Dynein localization shown in reconstructed confocal Z-stacks of cells stained with DAPI (blue) and anti-dynein antibodies (Alexa Fluor 549 secondary antibody, red). Scale bar = 3μm (x1,000 original magnification). **(D)** Dynein immunoblot of infected and uninfected cell lysates at 0, 8, and 24 h timepoints and the loading control GAPDH.

Cytoplasmic dynein is a motor protein that mediates retrograde transport along MTs and contributes to centrosome and nuclear positioning (31). We show here that a significant reduction in nuclear hypersegmentation was observed in Hp-PMNs treated with the dynein AAA^+^ ATPase inhibitor ciliobrevin D (32), whereas nuclear morphology of the uninfected control PMNs was unchanged (Figures 5A,B). Confocal microscopy revealed that although dynein was diffusely distributed throughout control PMNs, in agreement with published data (33), it appeared more abundant on nuclei of Hp-PMNs (Figure 5C). Nonetheless, total dynein levels did not change with infection or across time (Figure 5D). Thus, dynein activity and potentially protein relocalization contributed to hypersegmentation in our system.

### Lamin B Receptor and Lamin B1 Are Distributed Throughout the Nuclear Envelope

**Figure 6:**
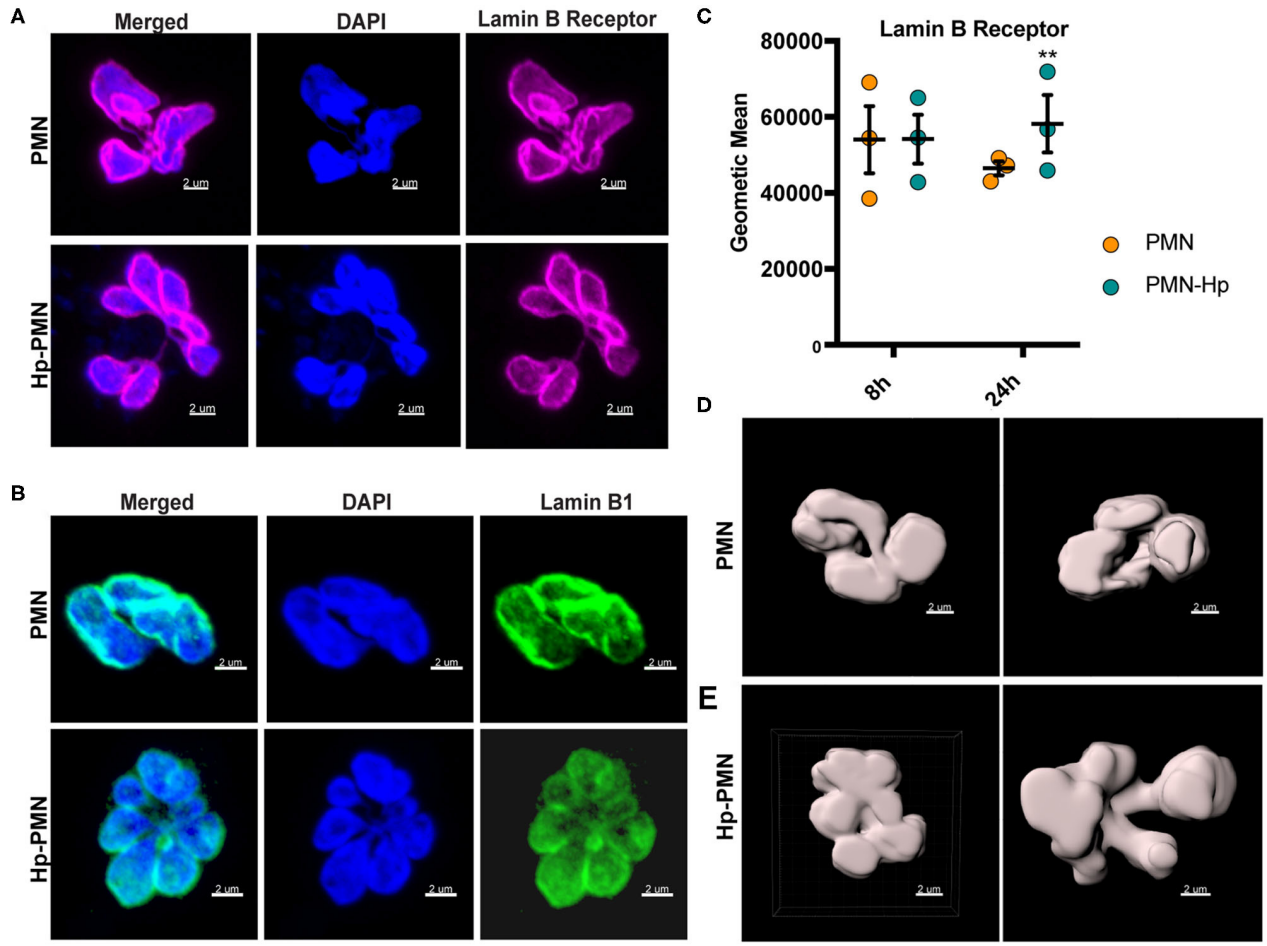
*H. pylori* has no apparent effect LBR and lamin B1 localization or LBR abundance. **(A,B)** LBR **(A)** and Lamin B1 **(B)** confocal Z-stack reconstructions of uninfected and infected PMNs at 24 h. DAPI was used to detect DNA (blue), Alexa Fluor 647-conjugated secondary antibodies were used to visualize LBR (magenta), Alexa Fluor 488-conjugated secondary antibodies were used to visualize lamin B1 (green). **(C)** LBR in control and infected PMNs at 8 and 24 h was quantified using flow cytometry. Geometric mean fluorescence and standard error of the mean are shown. Two-way ANOVA and Tukey's multiple comparison test were used to determine significance. **P ≤ 0.01, *n* = 3. **(D,E)** LBR STED reconstruction and nuclear periphery tracing for surface rendering used to quantify nuclear volume in Figure 7. **(D)** Two views of a single uninfected PMN. **(E)** Two different infected cells. Scale bar = 2 μm (x1,000 original magnification).

LBR is an abundant multi-spanning integral protein of the inner nuclear membrane that binds directly to intermediate filament proteins of the lamin B family and also interacts with heterochromatin, thereby anchoring the lamina and DNA to the membrane (18, 34). Confocal imaging demonstrated that both LBR and lamin B1 were distributed throughout the nuclear envelopes of control as well as Hp-PMNs (Figures 6A,B) and similar results were obtained for lamin B2 (data not shown). It is established that LBR levels increase during neutrophil development and that *LBR* gene dosage directly influences the abundance of this protein and PMN nuclear morphology (34). Specifically, neutrophils from people with Pelger-Hüet anomaly have only one functional copy of *LBR* and are hyposegmented (bi-lobed), whereas human PMNs with three functional *LBR* alleles are hypersegmented (34). With this in mind, we quantified LBR using flow cytometry (Figure 6C) and found that control and infected cells were indistinguishable at 8 h. At 24 h, LBR was slightly elevated in Hp-PMN and slightly reduced in control PMNs, likely because some of the latter cells were apoptotic (16) (Figures 1B, 4B, 5B), but these changes were not significant relative to the 8 h baseline. As LBR traffics to the nuclear membrane from the ER (18), we also examined this organelle using antibodies to the marker protein PDI but detected no discernable differences between Hp-PMN and the uninfected controls (Supplementary Figure 3).

### Microtubule-Dependent Nuclear Enlargement Precedes Hypersegmentation

**Figure 7:**
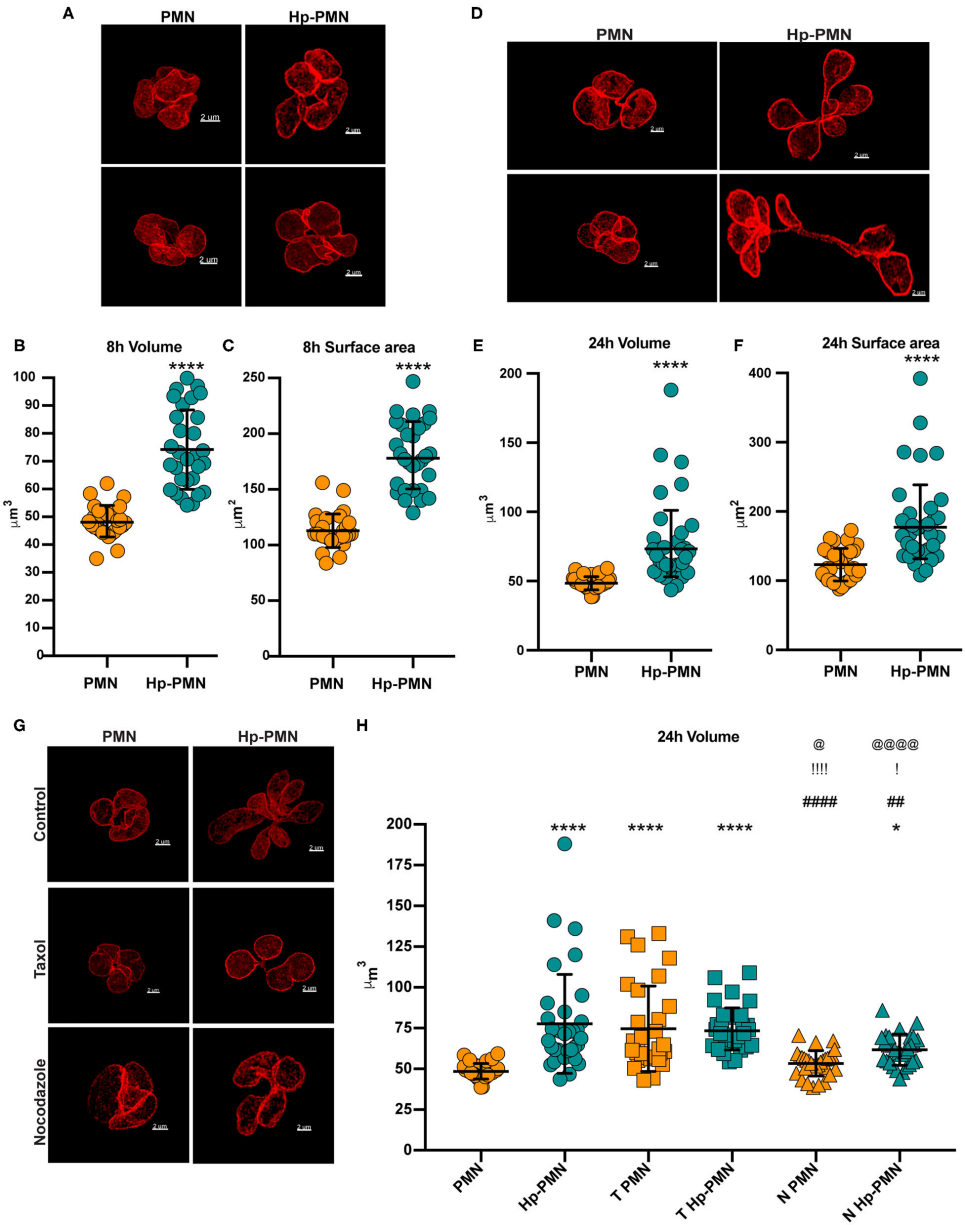
*H. pylori* infection and microtubule dynamics alter nuclear volume. Uninfected and infected PMNs were stained to detect LBR (red) after 8 h **(A–C)** or 24 h **(D–H)**. As indicated in **(G,H)**, cells were also treated with taxol (T) or nocodazole (N) at time zero. **(A,D,G)** Representative 3D reconstructions of STED Z-stacks. Scale bar = 2μm (x1,000 original magnification). Quantification of nuclear volume **(B,E,H)** and surface area **(C,F)** were done by tracing the nuclear periphery on each stack using the volume Imaris extension as shown in Figure 6D (see also Supplementary Videos 5, 6). Ten cells were analyzed per donor, per condition. *n* = 3. Graphs show the mean ± SD along with data for individual cells. Statistical significance was determined by two-way ANOVA and Tukey's multiple comparison test. ****P ≤ 0.0001 and *P ≤ 0.05 vs. PMN. ^####^*P* ≤ 0.0001 and ^##^*P* ≤ 0.01 vs. Hp-PMN. ^!!!!^*P* ≤ 0.0001 and ^!^*P* ≤ 0.05 vs. T- PMN. ^@@@@^*P* < 0.0001, ^@^*P* ≤ 0.05 vs. T Hp-PMN.

Next, we stained cells to detect LBR and utilized STED super-resolution microscopy to obtain detailed analyses of nuclear size and shape at 8 and 24 h. Volume and surface area were measured on Z-stack reconstructions after surface rendering, depicted in Figures 6D,E and Supplementary Videos 3–6. Uninfected PMN nuclei were very similar to one another in morphology (Figures 7A,D), volume (Figures 7B,E), and surface area (Figures 7C,F) at both time points and for all donors tested. However, it is important to note that only segmented control cells were included in this analysis and cells with condensed apoptotic nuclei were excluded. In marked contrast, the morphology of infected PMN nuclei was very heterogeneous, in agreement with the data shown in Figures 1A, 4A, 5A, 6A,B and our previous work (16). Moreover, significant increases in nuclear volume and surface area were observed in Hp-PMNs as early as 8 hpi (Figures 7B,C) and were sustained to at least 24 hpi (Figures 7E,F). As we have shown that only a small fraction of cells (~15%) are hypersegmented at 8 hpi (16), these new data demonstrate that changes in nuclear volume and surface area preceded hypersegmentation and were sustained thereafter, and as such are defining characteristics of Hp-PMNs.

Finally, we examined the effects of MTs on nuclear volume at 24 h, performing similar analyses of control and infected cells treated with nocodazole or taxol at time zero (Figures 7G,H) and with comparison to the no-drug controls (Figures 7E,G,H). The data indicate that bacteria-driven nuclear enlargement was nearly ablated by nocodazole (Figures 7G,H). On the other hand, taxol treatment increased nuclear volume to a similar extent as *H. pylori* infection (Figures 7G,H) despite its inability to induce hypersegmentation (Figures 4A,B), and these two treatments had no additional effect on nuclear volume when used in combination. Altogether, our data demonstrate that PMN nuclei enlarged significantly prior to hypersegmentation, that these two processes can be uncoupled, and that they depended on MTs to different extents.

## Discussion

Relatively little is known about the mechanisms that control neutrophil nuclear morphology. Until recently, PMN hypersegmentation was believed to be a rare event that arose primarily in the context of folate or vitamin B12 deficiency or as a consequence of mutations leading to triplication of *LBR* (18, 34). However, since the discovery of PMN plasticity it has become apparent that hypersegmentation is a defining feature of neutrophils in many microenvironments and disease states, including several types of cancer and acute as well as chronic inflammation (5, 9–12, 15, 16). Nonetheless, how hypersegmentation is achieved and the functional consequences of this unusual nuclear morphology are unknown. The results of this study provide significant insight into the molecular mechanisms that mediate hypersegmentation of mature human neutrophils during *H. pylori* infection. Our data reveal a prominent role for MT dynamics and dynein activity in this process and clearly show that hypersegmentation can be achieved without changes in DNA content or LBR abundance.

In most non-dividing cells MTs are relatively stable with half-lives of 5–20 min (35). In marked contrast, neutrophil MTs are highly dynamic, exhibit extensive tyrosination, and have a calculated half-life of only 15–30 s (35). Herein, we demonstrated that dynamic, tyrosinated MTs increased markedly in number and length after *H. pylori* infection. Sensitivity to inhibition by nocodazole and taxol at early and late time points demonstrated that MT dynamics were required to induce and maintain hypersegmentation, and accelerated MT rearrangement following taxol removal suggests that MT dynamics were enhanced. Z-stack images and videos show that MTs emanating from the MTOC extended between and around nuclear lobes of both control and infected neutrophils. Nevertheless, we also show that MTs are not required to sustain the normal, lobed morphology of mature PMNs, in agreement with published data (35). These data reinforce the fact that mechanisms of segmentation and hypersegmentation are distinct.

The ability of MTs to exert force on the nucleus is established and the results of this study support a model of hypersegmentation driven by mechanotransduction across the nuclear envelope. MTs interact with nuclei by one of two mechanisms. Typically, MTs bind directly to LINC (Linker of Nucleoskeleton and Cytoskeleton) complex proteins in the nuclear membrane which are, in turn, connected to lamin A, its splice variant lamin C and chromatin. As nuclei of most cells are relatively rigid, force exerted by MTs is utilized for nuclear rotation and positioning (18, 36). During neutrophil development lamin A/C and essential components of the LINC complex are downregulated and disappear, and therefore cannot account for hypersegmentation in our system despite the central role of lamin A/C downregulation in nuclear deformability (18).

Dynein is a motor protein and AAA^+^ ATPase that mediates retrograde transport and also allows MTs to exert force on other cellular structures. At the onset of mitosis, dynein is recruited to the surface of prophase nuclei and in this locale regulates focal polymerization of MTs that invaginate the nuclear envelope adjacent to the MTOC and exert a pulling force that culminates in catastrophic membrane rupture (37). Although the essential role of dynein in mitosis is unequivocal, its ability to interact with interphase nuclei has only recently been described (38, 39). It has been proposed that dynein may interact preferentially with tyrosinated MTs, and in melanoma cell lines nocodazole enhances dynein nuclear accumulation (38, 39). In contrast, we show here that dynein is distributed diffusely throughout human PMNs, confirming results of Huang et al. (33), yet accumulated at the nucleus after *H. pylori* infection. As dynein activity and MT dynamics were required for hypersegmentation, we propose that processes typically utilized for nuclear envelope breakdown in mitosis may be repurposed to induce hypersegmentation during *H. pylori* infection. Notably, dynein connects MTs to nuclei not *via* LINC complexes but rather *via* interactions with nuclear pore complexes and Ran binding protein 2 (40). Mechanisms that govern the magnitude and duration of dynein accumulation on interphase nuclei remain to be determined.

In the context of this study, we used STED super-resolution microscopy and LBR staining to analyze neutrophil nuclear morphology in unprecedented detail and our data demonstrate that nuclei enlarged significantly prior hypersegmentation. Model systems commonly used for studies of nuclear size regulation include cancer cells, *Xenopus* oocytes, yeast, plants, neurons, fibroblasts and several cell lines (41–44). Although the mechanisms are only partially understood, it is clear that chromatin organization and the magnitude of nuclear import are critical players (41, 42). Conversely, DNA abundance is largely non-contributory, and roles for MTs are context and cell type-specific (41–43). For example, MTs constrain nuclear size in mesenchymal stem cells, have no effect on L929 cells, and collaborate with dynein for nuclear expansion in *Xenopus* oocytes (43, 44). At a minimum, MTs can influence nuclear size by delivering cargo to the import machinery in the vicinity of nuclear pores and by exerting force on the nuclear envelope that can, in and of itself, alter chromatin structure and change gene expression (39, 40, 45). Although similar studies of neutrophils have not been performed, it is of interest that heterochromatin, which is abundant in PMNs, is tethered to the lamina by LBR whereas euchromatin interacts with nuclear pore complexes (18). Whether chromatin organization or nuclear transport pathways are modulated in our system remains to be determined, but data presented here show that bacteria-triggered nuclear enlargement was inhibited by nocodazole and can be recapitulated by taxol in the absence of infection; and our published data show that gene expression and protein synthesis are required for hypersegmentation (16). Thus, it is attractive to predict that transcriptional reprogramming as well as nuclear size and morphology may be inherently linked at the level of MT-driven mechanotransduction. Elucidating the effects of *H. pylori* on neutrophil gene expression, chromatin structure and nuclear transport are areas of active investigation in our laboratory.

The significance of hypersegmentation with respect to neutrophil function and fate, and whether other bacterial pathogens induce a similar phenotype are unknown. It is often stated that a segmented nucleus may facilitate neutrophil migration through tight spaces (18, 46, 47). Accordingly, we are using a multifaceted approach to elucidate effects on hypersegmentation on neutrophil migration, and preliminary results suggest that this process is impaired rather than enhanced (Prichard et al., unpublished data). Thus, it is conceivable that hypersegmentation together with extended lifespan may contribute to PMN accumulation in the gastric mucosa during *H. pylori* infection. We are also actively pursuing the possibility that nuclear enlargement and hypersegmentation may be hallmarks of chromatin reorganization and changes in gene expression occurring on a scale greater than is typically observed in neutrophils and which may, in turn, influence PMN immunoregulatory properties as well as the mode and efficiency of PMN death. To our knowledge, hypersegmentation has not yet been reported in the context of other infections, and it will be important to determine if this phenotype is specific for *H. pylori*. To this end, we have shown that *Francisella tularensis* does not alter neutrophil nuclear morphology despite its ability to replicate in the cytosol and prolong cell lifespan (23), and pilot studies by our group suggest that hypersegmentation does not occur in the context of neutrophil infection with *Neisseria gonorrhoeae* or *Staphylococcus aureus*. Additional studies of viral, bacterial, fungal, and parasitic pathogens are clearly needed, and it is tempting to speculate that nuclear morphology may receive more attention in the future as one aspect of the rapidly expanding neutrophil plasticity field.

In summary, we identified a new mechanism of neutrophil hypersegmentation that is driven by MT dynamics and dynein. To achieve this, we capitalized on the tractability of our *in vitro H. pylori* infection model and it will of interest in future studies to determine if this mechanism is conserved in other hypersegmented neutrophil populations and subsets. Based on our results, we propose that dynein is responsible for connecting MTs to the nucleus and that MTs interacting with dynein force the enlarged nucleus to hypersegment *via* mechanotransduction across the membrane and by repurposing molecular mechanisms that elicit nuclear rupture at the onset of mitosis in other cell types. Whether these processes are coupled to chromatin rearrangements or are essential for neutrophil transcriptional reprogramming during *H. pylori* infection remain to be determined. In future studies it will also be important to determine if the nuclear morphology of individual cells is static or dynamic, to identify sources of membrane used for nuclear expansion, and to investigate signals that modulate MT abundance.

## Data Availability Statement

The raw data supporting the conclusions of this article will be made available by the authors, without undue reservation.

## Ethics Statement

The studies involving human participants were reviewed and approved by Institutional Review Board of the University of Iowa. The patients/participants provided their written informed consent to participate in this study.

## Author Contributions

L-AHA conceived of the study, designed experiments, analyzed data and co-wrote the manuscript. SLS-DT designed and performed experiments, analyzed data and co-wrote the manuscript. All authors contributed to the article and approved the submitted version.

## Conflict of Interest

The authors declare that the research was conducted in the absence of any commercial or financial relationships that could be construed as a potential conflict of interest.
