# Supplementary material for: Microtubules and Dynein Regulate Human Neutrophil Nuclear Volume and Hypersegmentation During H. pylori Infection
Source: Front Immunol. 2021 Mar 22;12:653100. doi: 10.3389/fimmu.2021.653100 (PMC8019731; doi:10.3389/fimmu.2021.653100)
Supplement: Supplementary file 1 [file Data_Sheet_1.PDF]

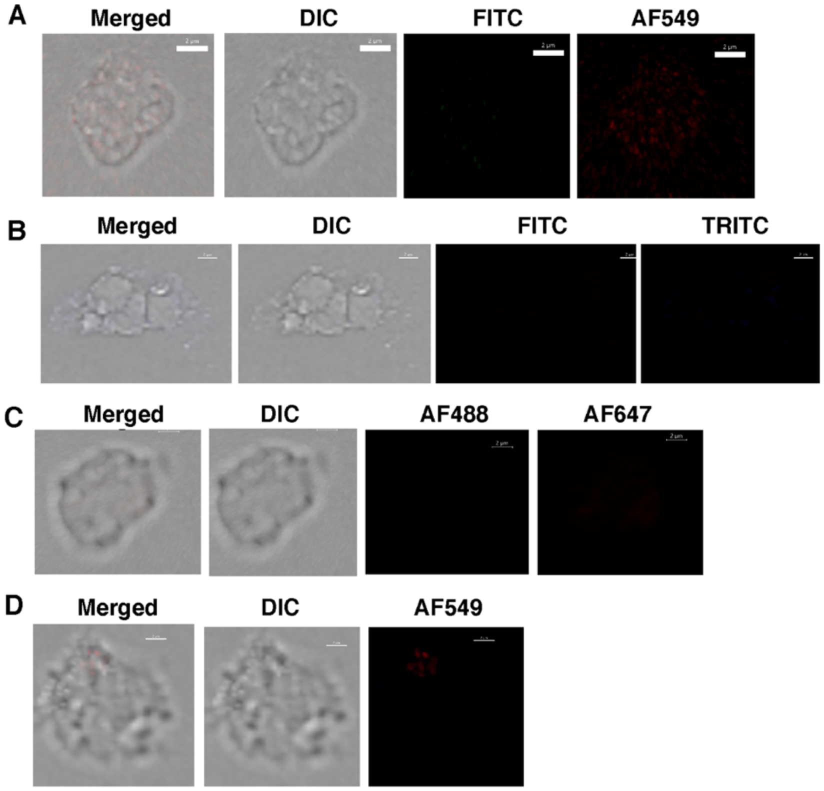

Supplemental Figure 1. Secondary antibody only staining. Confocal images show the extent of background staining obtained when cells were fixed and permeabilized at 24 h via methods used to detect microtubules and dynein (A and B), lamin B receptor and lamin B1 (C), or ER (D) and stained with secondary antibodies alone. F(ab')$_2$ antibodies were conjugated to FITC, TRITC, or Alexa Fluor (AF) 488, 549, or 647 as indicated. Images were processed using Imaris software. Fluorescence, differential interference contrast (DIC) and merged images are shown. Scale bar = 2μm. (x630 original magnification).

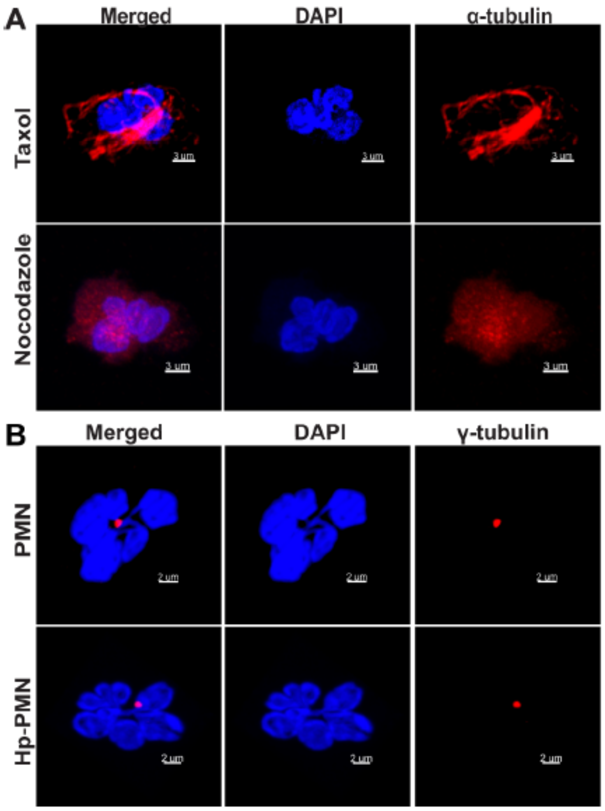

Supplemental Figure 2. Efficacy of nocodazole and taxol and imaging of γ-tubulin at the MTOC. (A) Confocal images confirm taxol-induced bundling and nocodazole-induced depolymerization of microtubules at 24 h. Cells were stained with DAPI (blue) along with anti-α-tubulin and secondary antibodies conjugated to Alexa Fluor 549 (red). Scale bar = 3μm (1,000x original magnification). (B) No centrosome splitting was observed in infected or uninfected PMNs at 24 h. Cells were stained with DAPI (blue) and secondary Alexa Fluor 549 secondary antibodies (red) were used to visualize γ-tubulin. Scale bar = 2μm (1,000x original magnification).

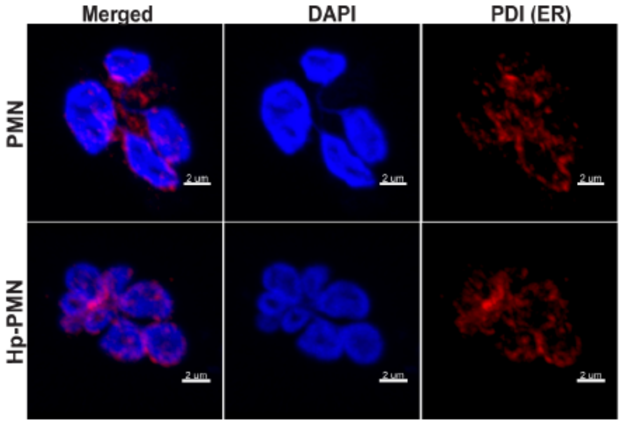

Supplemental Figure 3. Infection does not alter the appearance of the ER. Confocal Z-stack reconstructions of control and *H. pylori*-infected PMNs at 24 h that were stained to show DNA (DAPI, blue) and the ER marker PDI (Alexa Fluor 549-conjugated secondary antibodies, red). Images are representative of three experiments. Scale bar = 2µm

Supplemental Video Legends

Video 1. Representative FITC-tyrosinated-α-tubulin STED Z-stack reconstruction of a control uninfected neutrophil at 24 hours.

Video 2. Representative FITC-tyrosinated-α-tubulin STED Z-stack reconstruction of an *H. pylori*-infected neutrophil at 24 hours after infection.

Video 3. Representative STED Z-stack reconstruction of uninfected neutrophil at 24 hours stained to show lamin B receptor (TRITC-conjugated secondary antibody, red).

Video 4. Representative STED Z-stack reconstruction of an *H. pylori*-infected neutrophil at 24 hours after infection stained to show lamin B receptor (TRITC-conjugated secondary antibody, red).

Video 5. Representative control neutrophil at 24 hours stained to detect lamin B receptor (TRITC, red) and showing nuclear periphery tracing used to determine volume and surface area.

Video 6. Representative *H. pylori*-infected neutrophil at 24 hours after infection stained to detect lamin B receptor (TRITC, red) and showing nuclear periphery tracing used to determine volume and surface area.
